# Relating diffusion tensor imaging measurements to microstructural quantities in the cerebral cortex in multiple sclerosis

**DOI:** 10.1002/hbm.24711

**Published:** 2019-07-29

**Authors:** Rebecca McKavanagh, Mario Torso, Mark Jenkinson, James Kolasinski, Charlotte J. Stagg, Margaret M. Esiri, Jennifer A. McNab, Heidi Johansen‐Berg, Karla L. Miller, Steven A. Chance

**Affiliations:** ^1^ Nuffield Department of Clinical Neurosciences University of Oxford Oxford United Kingdom; ^2^ Wellcome Centre for Integrative Neuroimaging, FMRIB, Nuffield Department of Clinical Neurosciences University of Oxford Oxford United Kingdom

**Keywords:** cortex, diffusion tensor imaging, minicolumns, multiple sclerosis, postmortem

## Abstract

To investigate whether the observed anisotropic diffusion in cerebral cortex may reflect its columnar cytoarchitecture and myeloarchitecture, as a potential biomarker for disease‐related changes, we compared postmortem diffusion magnetic resonance imaging scans of nine multiple sclerosis brains with histology measures from the same regions. Histology measurements assessed the cortical minicolumnar structure based on cell bodies and associated axon bundles in dorsolateral prefrontal cortex (Area 9), Heschl's gyrus (Area 41), and primary visual cortex (V1). Diffusivity measures included mean diffusivity, fractional anisotropy of the cortex, and three specific measures that may relate to the radial minicolumn structure: the angle of the principal diffusion direction in the cortex, the component that was perpendicular to the radial direction, and the component that was parallel to the radial direction. The cellular minicolumn microcircuit features were correlated with diffusion angle in Areas 9 and 41, and the axon bundle features were correlated with angle in Area 9 and to the parallel component in V1 cortex. This may reflect the effect of minicolumn microcircuit organisation on diffusion in the cortex, due to the number of coherently arranged membranes and myelinated structures. Several of the cortical diffusion measures showed group differences between MS brains and control brains. Differences between brain regions were also found in histology and diffusivity measurements consistent with established regional variation in cytoarchitecture and myeloarchitecture. Therefore, these novel measures may provide a surrogate of cortical organisation as a potential biomarker, which is particularly relevant for detecting regional changes in neurological disorders.

**Abbreviations**
AngleR: angle between the principal diffusion direction and the radial minicolumn direction across the cortex
DTI: diffusion tensor imaging
GM: grey matter
MD: mean diffusivity
ParlPD: the component of the principal diffusion vector that was parallel to the radial minicolumn direction across the cortex
PDD: principal diffusion direction
PerpPD: the component of the principal diffusion vector that was perpendicular to the radial minicolumn direction across the cortex
PLP: proteolipid protein
PMI: postmortem interval
RGB: red, green, blue
ROI: region of interest
SI: scan interval
TE: echo time
TR: relaxation time
V1: primary visual cortex
WM: white matter

## 1 INTRODUCTION

Magnetic resonance imaging (MRI) is increasingly used to visualise the detailed structure of the cerebral cortex (e.g., cortical layers (Barazany & Assaf, 2011; Fatterpekar et al., 2002)). In particular, recent diffusion MRI studies have begun to investigate the diffusion signal in cortex (Anwander, Pampel, & Knosche, 2010; Cohen‐Adad et al., 2012; Hasan et al., 2007; Heidemann et al., 2009; Jeon et al., 2012; Jespersen, Leigland, Cornea, & Kroenke, 2012; Kang, Herron, Turken, & Woods, 2012; Kleinnijenhuis et al., 2013; Leuze et al., 2011; Leuze et al., 2012; McNab et al., 2013; Vrenken et al., 2006). This study aims to investigate correlations between cortical DTI and histology, with the long‐term goal of developing a biomarker that can detect pathological differences, which often rely on patterns of cytoarchitectural disruption across brain regions. Although diffusion in cortex exhibits some degree of fractional anisotropy (FA) (Anwander et al., 2010; Heidemann et al., 2009), most attention has been paid to the mean diffusivity (MD), which changes with age, pathology and across brain regions (Jeon et al., 2012).

Several recent studies have shown that diffusion in the cortex is largely radial (i.e., perpendicular to the cortical surface) (McNab et al., 2009) while also showing regional variation (Anwander et al., 2010; Kang et al., 2012; Kleinnijenhuis et al., 2013; McNab et al., 2013) and this has been interpreted as reflecting the cortex's anisotropic cytoarchitecture (Jespersen et al., 2012; Leuze et al., 2012). Such investigations have assessed the principal diffusion direction (PDD), revealing areas of consistently radial diffusion in motor (Anwander et al., 2010; McNab et al., 2013) and prefrontal (Anwander et al., 2010) cortex, and mixed reports of either more tangential (parallel to the cortical surface) (McNab et al., 2013) or radial (Kang et al., 2012) diffusion in other areas, for example, Heschl's gyrus.

We have previously applied postmortem (PM) diffusion imaging to the investigation of the relationship between cortical histology measures and white matter diffusion properties (Kolasinski et al., 2012). The target was white matter tracts in multiple sclerosis, based on the established sensitivity of MD and FA to demyelination and other aspects of white matter degeneration (Beaulieu, 2002; Schmierer et al., 2008). Measurements in the white matter tracts between cortical and subcortical grey matter areas correlated with standard histological measures in those areas (cortical thickness and cell density), implicating Wallerian degeneration (Kolasinski et al., 2012). Given the increasing interest in the cerebral cortex in multiple sclerosis (Wegner, Esiri, Chance, Palace, & Matthews, 2006), we have extended this investigation of the relationship between DTI and histology to the grey matter of the cortex in the same cohort.

Therefore, the present study aimed to identify variation in histological measurements of the radial elements of cortical structure corresponding to the diffusion signal. A fundamental structural unit of the cortex is the minicolumn, a vertical string of neurons, with associated dendrites and myelinated axon bundles (Buxhoeveden & Casanova, 2002; Casanova, Konkachbaev, Switala, & Elmaghraby, 2008; Mountcastle, 1997). Although it may be expected that aspects of the axonal bundles (including membranes and myelination) descending from Layers III to VI are a significant contributor to diffusion in the cortex, the bulk of histological literature on cortical radial organisation depends on measurement of the minicolumns as assessed by Nissl staining of the cell bodies. This literature documents regional differences (Chance et al., 2011) and effects of both ageing (Chance et al., 2011; Chance, Casanova, Switala, Crow, & Esiri, 2006; Di Rosa, Crow, Walker, Black, & Chance, 2009) and pathology in other disorders (Casanova, Buxhoeveden, Switala, & Roy, 2002). Previous work has demonstrated almost identical spacing between the axon bundles and the minicolumns (Casanova et al., 2008) supporting the idea that both are measuring different aspects of the same structure. Both cellular and axonal components were assessed in the present study and their correspondence to MRI diffusion measures was also examined. One recent study has reported on the quantitative relationship between FA and axon orientation in the cerebral cortex in multiple sclerosis (Preziosa et al., 2019). Here, we explored additional diffusivity metrics not previously reported in the cortex and their relationships with axon bundles and minicolumn structure in multiple sclerosis. Water molecules move approximately 10 μm during a typical MR measurement time (Mori & Zhang, 2006) which is about one‐third the width of a minicolumn. Although DTI is a relatively crude tool for analysing diffusion MRI, which is not well suited to modelling relationships with the underlying anatomy, it can nonetheless be useful for exploring markers of disease by virtue of its simplicity and wide applicability to a large variety of acquisition protocols, especially those common in the clinical realm.

This study set out to investigate the hypothesis that variation in the principal diffusivity relates to aspects of the best known radial cortical elements, the minicolumn, and axonal bundle organisation. The study did not model or attempt to explore a deep interpretation of the cause of the relationship between histology and diffusivity, but was intended as a study to observe correlations and differences in disease that could be useful for developing biomarkers.

## 2 MATERIAL AND METHODS

### 2.1 Patients/samples

Fixed whole brains from nine multiple sclerosis patients (Table 1) were obtained from the UK MS Tissue Bank (Imperial College, Hammersmith Hospital Campus, London). Brains were stored in 10% formalin before being transferred to a perfluorocarbon solution (Fomblin LC08; Solvay Inc.; Bollate, Italy) for scanning, which contributes no MRI signal and provides susceptibility matching to tissue (reducing image artefacts).

**Figure 1:**
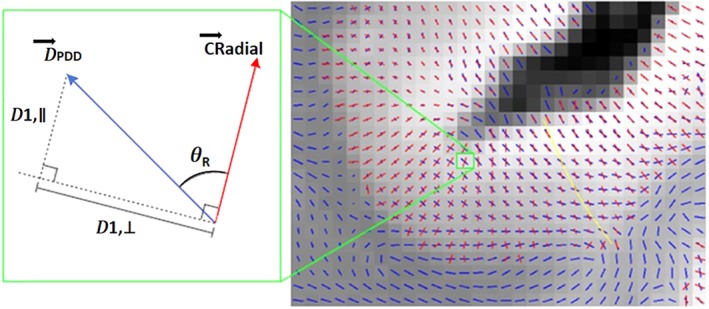
Example of the cortical diffusion data for one representative region (right), including an illustrative voxel example of the derived diffusion‐based measures (left). A blue line indicates the principal diffusion vector in a voxel: on the right, only the direction is indicated, while on the left, the diffusion tensor component along the principal diffusion direction (PDD) vector (*D*_PDD_) is shown. A red line indicates the radial direction within the cortex (CRadial). The angle of radiality, AngleR (notation *θ*_R_), in a voxel is the angle between the red and blue lines. The perpendicular diffusivity, PerpPD (notation 𝐷1,⊥), was calculated by projecting *D*_PDD_ onto the plane perpendicular to CRadial. The parallel diffusivity, ParlPD (notation 𝐷1,∥), was calculated by projecting *D*_PDD_ onto the CRadial. Quantities were averaged along the radial cortical profile across the cortical layers, reflecting the minicolumnar organisation, as indicated for a set of voxels by the yellow line

**Table 1:** Characteristics of brains provided for study. MS cases and HCs

Subject	Sex	Age	Hemisphere	Disease progression	Disease duration (years)	Time disease was progressive (years)^a^	Time in a wheelchair (years)^a^	PMI (h)	Scan interval (days)	Cause of death
MS 254	F	69	R	Secondary	37	12	7	66	1,198	MS
MS 281	F	74	L	Primary	33		17	40	929	Sepsis
MS 314	F	78	R	Secondary	45	24	17	60	435	Colonic carcinoma
MS 316	F	79	R	Secondary	55	40	36	26	1,052	Pneumonia
MS 322	M	72	L	Secondary	28	4		59	1,201	Pneumonia
MS 332	F	50	R	Secondary	22	10	2	69	1,134	Breast cancer mets
MS 334	M	66	R	Secondary	15		1	37	1,126	Prostate cancer
MS 396	F	86	R	Primary	54			54	578	Lymphoma
MS 400	F	60	L	Secondary	11		7	21	539	MS
HC 1	M	72	R	–	–	–	–	24	693	
HC 2	F	88	R	–	–	–	–	24	655	
HC 3	M	68	R	–	–	–	–	48	1,236	
HC 4	F	82	L	–	–	–	–	48	1,197	
HC 5	F	68	L	–	–	–	–	48	1,216	Pancreas carcinoma
HC 6	F	48	R	–	–	–	–	48	1,151	Pneumonia

Abbreviations: HC, healthy control; MS, multiple sclerosis; PMI, postmortem interval.

a MS clinical details where data were not available for all cases.

### 2.2 MRI scanning

Nine multiple sclerosis patients and six control brains from a pre‐existing cohort in the Oxford Brain Bank, were used for the MRI comparison.

Scanning was carried out on a Siemens Trio 3T scanner using a 12‐channel head coil. Scanning was conducted at room temperature and each scan session lasted approximately 24 h. Diffusion weighted data were acquired using a modified spin‐echo sequence with three‐dimensional (3D) segmented EPI (TE/TR = 122/530 ms, bandwidth = 789 Hz/pixel, matrix size: 168 × 192 × 120, resolution 0.94 × 0.94 × 0.94 mm^3^). Diffusion weighting was isotropically distributed along 54 directions (*b* = 4,500 s/mm^2^) with six *b* = 0 images. This protocol takes approximately 6 h, and three averages were acquired for 18 h total diffusion imaging. Structural scans were acquired using a 3D balanced steady‐state free precession sequence (TE/TR = 3.7/7.4 ms, bandwidth = 302 Hz/pixel, matrix size: 352 × 330 × 416, resolution 0.5 × 0.5 × 0.5 mm^3^). Images were acquired with and without RF phase alternation to avoid banding artefacts. This was averaged over eight repeats to increase signal to noise ratio (SNR). For more details, see Miller et al. (2011).

Data were processed using the FMRIB software library (FSL) (Smith et al., 2004; Woolrich et al., 2009). The FSL diffusion toolbox was used to process diffusion weighted data, which incorporates an in house processing pipeline to compensate for gradient‐induced‐heating drift and eddy‐current distortions, to produce maps of FA, MD, and the diffusion tensor components (Miller et al., 2011).

### 2.3 Selection of brain regions

Measures of cortical thickness in dorsolateral prefrontal cortex (Area 9) and primary visual cortex (V1) and diffusion measures of connected white matter tracts (FA and MD) were correlated with histological myelination measures in our previous study (Kolasinski et al., 2012) and, as multiple sclerosis is a demyelinating disorder, these areas were chosen for further investigation in the present study. In addition, these areas are well characterised and are known to represent a range of cortical cytoarchitectural arrangements (i.e., wider minicolumns in Area 9 and narrower minicolumns in V1). An additional comparison region was included—the primary auditory cortex within Heschl's gyrus (Area 41)—because its columnar architecture is well characterised but there have been inconsistencies in previous reports on its PDD in healthy subjects (Kang et al., 2012; McNab et al., 2013). Investigation of multiple cortical regions allowed us to explore the sensitivity of measures of diffusion to regional differentiation, which would be of interest in future investigations of neurological disorders.

### 2.4 Neurohistological sampling

Brains were sectioned coronally and the diagnosis of multiple sclerosis was confirmed by a clinical neuropathologist. Blocks of size 25 × 25 × 10 mm^3^ were sampled for each of the three regions from one hemisphere per brain (a representative random sample of hemispheres: 7 left, 8 right). Blocks and the surrounding tissue were photographed using an Olympus C‐5050 digital camera for reference. Area 9 included the middle and superior frontal gyri bounded inferiorly at the paracingulate sulcus and inferior frontal sulcus. Area 9 blocks were sampled level with the anterior limit of the cingulate gyrus. Area 41 blocks incorporated Heschl's gyrus, bordered medially by the insula cortex and laterally by the planum temporale. V1 blocks were sampled along the calcarine fissure, level with the medium transverse occipital gyrus. Region of interest (ROI) selection was confirmed cytoarchitecturally in accordance with von Economo and Koskinas (1925).

Tissue blocks were embedded in paraffin wax and serially sectioned at 10 μm for the minicolumn analysis and quantification of myelin levels, and at 30 μm for the bundle measurements. Sections were stained with cresyl violet (CV; Thermo Fisher Scientific, Waltham, MA) for minicolumn analysis, anti‐proteolipid protein stain (AbD Serotec, Oxford, UK) (anti‐PLP) for light transmittance myelin quantification, and Sudan black, a myelin sensitive lipophilic dye, for measurement of axonal bundles.

### 2.5 Cortical diffusivity analysis

This was a ROI approach. Cortical ROIs corresponding to those sampled histologically were delineated using manually created masks on the structural PM images. By careful reference to photographic images of the physically cut coronal brain slice before and after the tissue block was removed, and the corresponding Nissl stained slide, the closest matching coronal slice of the structural MRI scan was identified. Cortical ROIs were masked over 15 coronal slices of the MRI image centred around this slice, taking care to include only grey matter voxels to avoid contamination from white matter or CSF. The limits of the cortical ROIs were determined by careful comparison with the photographic images and corresponding Nissl stained slide. Novel software scripts (M.K., University of Oxford, 2018; patent application WO2016162682A1; U.S. patent application no. 15/564344) were used to generate cortical profiles on the MRI scans, that is, lines within the cortex in a radial direction, replicating the columnar organisation within the cortex. Values for the diffusion tensor derived measures were averaged along the cortical profiles, throughout the masked ROI, excluding the terminal slices at the anterior and posterior ends of the ROI. The measures calculated were MD, FA, and three measures relating to the principal diffusion component (see also patent application WO2016162682A1; U.S. patent application no. 15/564344), namely: the angle of the deviation between the radial direction and the PDD (AngleR, θ_R_); the principal diffusion component projected onto the plane perpendicular to the radial direction (described therefore as the perpendicular diffusivity, i.e., PerpPD, 𝐷1,⊥ [×10^−3^ mm^2^/s]), and the principal diffusion component projected onto the radial direction (described therefore as parallel to the radial direction, i.e., ParlPD, 𝐷1,∥ (×10^−3^ mm^2^/s) (Figure 1).

Averaging values reduced the influence of noise in the DTI data, effectively smoothing the data, and ensuring only directionality with some local coherence would dominate, guarding against the influence of random deflections from the radial direction. Averaging also provided consistency with the histological measurements, which similarly calculated a single value for each cortical region. Previous work has found that measures of the cytoarchitecture and myeloarchitecture are relatively stable within a cortical subregion (e.g., von Economo and Koskinas (1925)) indicating that it is valid to find an average value for that region.

### 2.6 Minicolumn analysis

Minicolumn width, based on cell bodies, was assessed in the histological tissue sections using a semiautomated procedure that has been described in detail previously (Buxhoeveden, Switala, Litaker, Roy, & Casanova, 2001; Casanova & Switala, 2005). This procedure gives a value for the minicolumn width consisting of the cell dense core region plus the associated neuropil space surrounding it. The neuropil spacing is the width of the cell sparse neuropil region between the cores of neighbouring minicolumns, while the core refers to the width of the cell dense region at the centre of the minicolumn. The microsegment number is the number of strings of cells that do not form a complete minicolumn because they are discontinuous with the rest of a minicolumn due to it passing out of the plane of section or due to minicolumn fragmentation as a result of pathology. Cell density refers to the density of cells recognised by the automated histology analysis programme within the field of view of each assessed digital photomicrograph (see Chance et al., 2011 for further discussion of microsegments and cell density).

For each ROI, three digital photomicrographs were taken from a single slide where possible, each containing a region of about 1 mm^2^. Image locations were selected using a random number generator, excluding areas of high curvature which have been shown to affect cell distribution (Chance, Tzotzoli, Vitelli, Esiri, & Crow, 2004). As minicolumns are clearest in Layer III, photographs were centred on that layer and obtained through a ×4 objective lens, with an Olympus BX40 microscope (more details can be found in Chance et al. (2004) and Di Rosa et al. (2009)). Values calculated from the three photographs were averaged to give a single value for each region.

### 2.7 Quantification of myelin levels

Cortical myelin content was assessed using light transmittance to quantify the intensity of myelin stain in anti‐PLP stained tissue sections. Data were collected using the AxioVision v4.7.2 software on a PC receiving a signal from an AxioCam MRc (Carl‐Zeiss, Jena, Germany) mounted on a BX40 microscope (Olympus, Japan) with a ×10 objective lens. The setup was calibrated in RGB mode with fixed white balance and incident light, using a standard slide/coverslip preparation and light filters (6, 25, and 100% transmittance). For each ROI, three measures of transmittance (T) were taken in different locations across Layers III–V using a 58,240 μm^2^ virtual frame on anti‐PLP stained sections and the resulting values averaged.

### 2.8 Axon bundle analysis

For each region, three photographs were obtained through a ×10 objective lens (resolution 1.10 μm) with an Olympus BX40 microscope, centred around Layer V as the axon bundles are clearest there. Areas of extreme curvature were avoided where possible, as was done for the minicolumn measurements.

**Figure 2:**
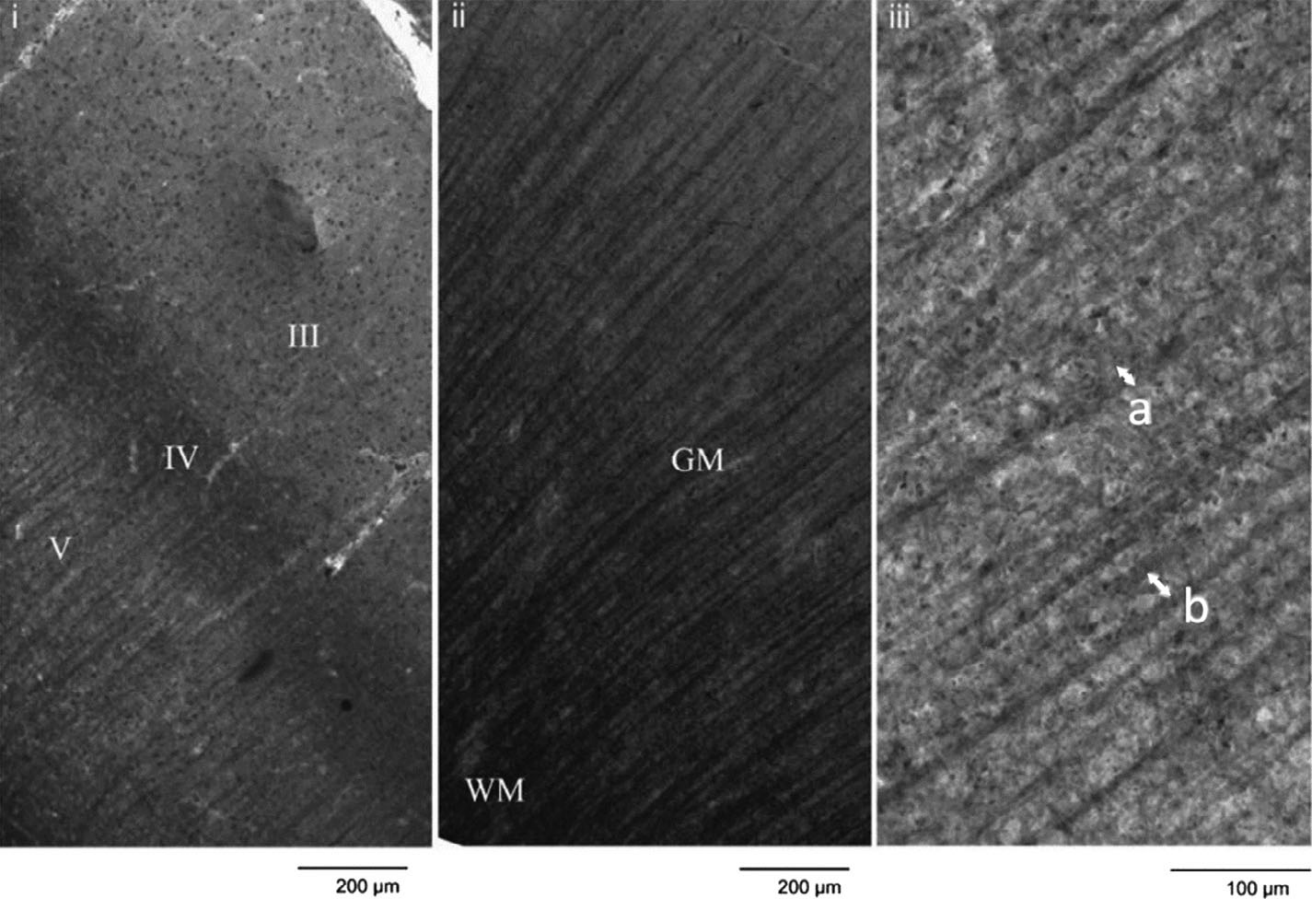
Sudan black stained section illustrating (i) cortical layers, (ii) tissue type, and (iii) measurements of axon bundle width (a) and axon bundle spacing (b), as indicated by the arrows [after McKavanagh, Buckley, & Chance, 2015]

Measurements of axon bundle centre‐to‐centre spacing, and the width of the bundles themselves were made manually in AxioVision, using the in‐built measurement tools (Figure 2). The digital resolution of the analysed images was 0.67 μm/pixel. A sample line of standard length (590 μm; determined by the size of the image view) was drawn across the centre of the photograph, perpendicular to the bundle direction in order to identify the bundles to be measured. Only bundles intersecting this line were measured, those that passed out of the plane of sectioning above or below the line were not included. Single axons or pairs of axons crossing the line were not considered to constitute axon bundles for the purposes of this analysis.

Bundles (>2 axons) were identified and their centres marked. Bundle spacing measurements were then made from the centre of each bundle marked in this way to the centre of the adjacent bundle. The width of each axon bundle was also measured. For the width measurements, the edges of the bundles were marked at the point where they intersected the line, and the bundle width was determined as the distance between these two points. Edges of axon bundles were distinguished by the change in intensity of staining from the background, which identified the start of the more darkly stained axon bundle. Pilot data revealed high reliability of this method, finding a high correlation (*r* = .737, *p* < .001) between measurements of photos taken on two different occasions. The values from the three photographs were then averaged to give a single value for bundle spacing and a single value for bundle width for each ROI.

This resulted in an average of 28 (±5), 22 (±5), and 44 (±5) bundles being sampled for Area 9, Area 41, and V1, respectively for each subject. It was not possible to assess the orientation of the axon bundles within the cortex in a manner directly comparable to our DTI analysis because such a 3D estimate is not possible in histological sections that have a limited depth, compounded by *z*‐direction compression on the microscope slide. However, taking a subset of cases with a relatively uncurved section of cortex where it may be assumed that the 3D geometric vertical is reasonably close to the two‐dimensional estimate from the histological section, we were able to measure the orientation of the axon bundles relative to this. This indicated that the axon bundles deviate from the radial direction across the cortex by an average of 3.50 (±2.68) degrees.

### 2.9 Statistical analysis

All data were analysed using SPSS v22 for Windows and the R statistical package (version 3.3.3) (R Core Team, 2013).

#### 2.9.1 Relationship between histology and DTI

The relationship between the microanatomy and MRI diffusion measures across the full data set was investigated by correlation analysis using Spearman's correlation coefficient. We carried out a correlation analysis for each of the three regions of interest (Area 9, Area 41, and V1) including the five diffusion measures (FA, MD, Angle_R, PerpPD, ParlPD) and the six histology measures (minicolumn width, core width, neuropil spacing, microsegment number, axon bundle width, bundle spacing). All *p* values were adjusted with false discovery rate (FDR) correction (FDR <0.05) (Benjamini & Yekutieli, 2001) and were reported using the approach of Preziosa et al. (2019) by providing *p* and *p*_FDR_ for significant results (see Table 5).

#### 2.9.2 Mean regional differences

Regional differences in both histology and DTI measures within groups were assessed using repeated measures analysis of variances (ANOVAs) and significant main effects were followed up with post hoc *t* tests. Regional differences in DTI between groups were assessed using repeated measures ANOVA.

#### 2.9.3 Histology measures

Relationships between the six histology measures (minicolumn width, core width, neuropil spacing, microsegment number, axon bundle width, bundle spacing) were investigated using Spearman's correlation coefficient and adjusted by FDR correction (FDR <0.05).

#### 2.9.4 Multiple sclerosis clinical correlates

Our previous study indicated a relationship between the degree of change in white matter and cellular organisation in Area 9 and V1 (Kolasinski et al., 2012). As disease duration was the only clinical measure available for all subjects (Table 1), the present study investigated whether there was a significant correlation between DTI derived measures and disease duration in these cortical regions (Area 9 and V1), and whether the correlations were different to that in the comparison region (Area 41). As age was expected to correlate with disease duration this was controlled for where appropriate using partial correlations using the standard SPSS recursive algorithm.

## 3 RESULTS

### 3.1 Comparison of DTI and histology measures in MS brains

**Figure 3:**
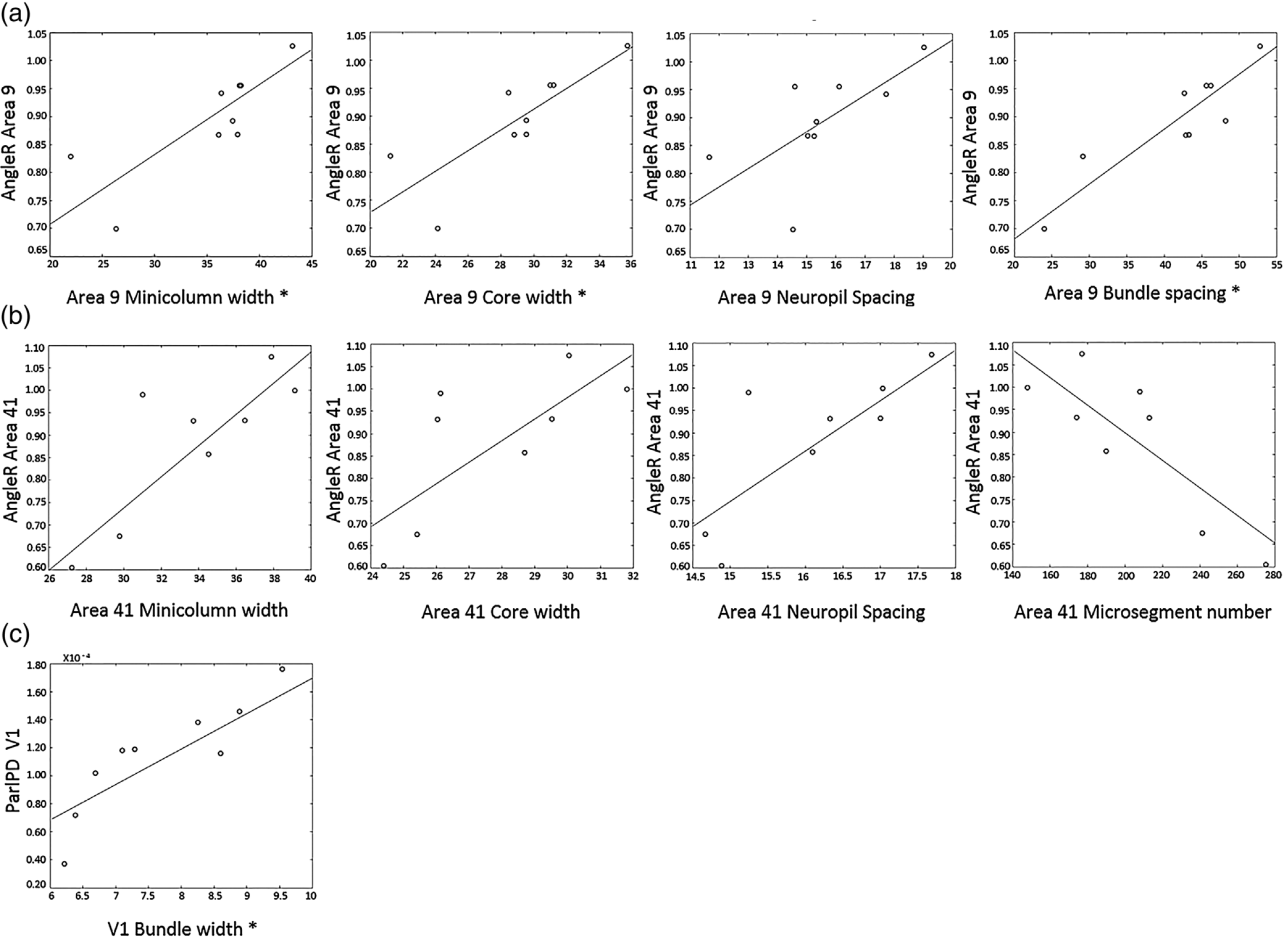
Specific relationships between DTI and histology in MS brains (relationships are shown with *p* value <.05; relationships with *P*_FDR_ < .05 surviving FDR correction are designated by * on the *x* axis)—See Section 4 for comments. AngleR values are expressed in radians (Θrad); ParlPD values are expressed in (×10^−3^ mm^2^/s)

None of the cortical diffusion measures were significantly affected by PM interval (PMI) or scan interval (SI). The analysis of interactions between cortical diffusivity and minicolumn organisation are reported in Table 5; (significant *p* values after FDR correction are reported as *p*_FDR_). In Area 9, Layer III showed significant direct correlations between AngleR and minicolumn width (*r* = .912, *p* = .001, *p*_FDR_ = .030), and between AngleR and core width (*r* = .879, *p* = .002, *p*_FDR_ = 0.045) (Figure 3 and Table 5). The positive relationship between AngleR and neuropil spacing did not survive FDR correction (*r* = .773, *p* = .024). Similar trends were also found in Area 41 between AngleR and minicolumn width (*r* = .817, *p* = .0013), core width (*r* = .849, *p* = .007), neuropil spacing (*r* = .833, *p* = .01), and microsegment number (*r* = −.786, *p* = .021) although these did not survive FDR correction (Table 5 and Figure 3). No associations between DTI measures and minicolumn indices were found in Area V1.

Concerning axon bundles, the correlation analysis revealed an association between AngleR and bundle spacing (*r* = .937, *p* = .000, *p*_FDR_ = .000) in Area 9 (Figure 3 and Table 5). No significant associations between AngleR and bundle measures were found in Area 41 and V1.

Finally, axon bundle width showed a significant direct correlation with ParlPD (*r* = .911, *p* = .001, *p*_FDR_ = .030) in Area V1 (Figure 3 and Table 5).

The relationship between cortical histology measures and the more commonly used DTI measure (MD) was not significant (see Table 5 for details).

### 3.2 DTI differences between groups and brain regions

**Figure 4:**
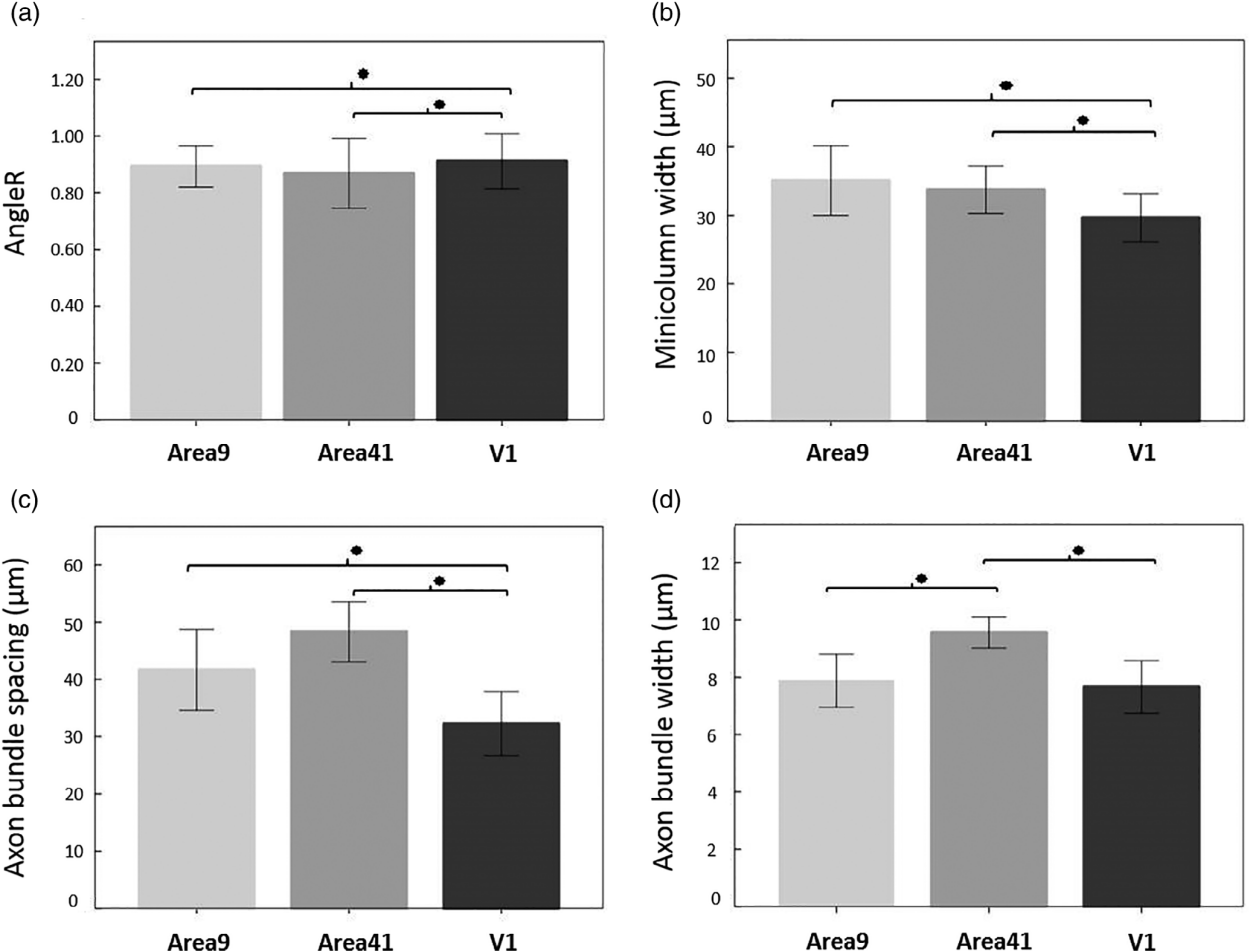
Regional differences in (a) AngleR, (b) minicolumn width, (c) axon bundle spacing, and (d) axon bundle width. Error bars show *SD*

We used a pre‐existent cohort of six controls to investigate the diffusivity measures between groups. Repeated measures ANOVAs revealed a significant main effect of diagnosis on diffusion measures, (Tables 2 and 3): AngleR (*F*(1,13) = 15.575, *p* = .002), MD (*F*(1,13) = 20.468, *p* = .002), PerpPD (*F*(1,13) = 39.177, *p* = .000), and ParlPD (*F*(1,13) = 16.905, *p* = .001) values were higher in multiple sclerosis cases compared to controls in all regions, while FA was not different between groups (*F*(1,13) = .928, *p* = .353). There was also a within subjects effect of region (Figure 4) due to higher AngleR values in V1 compared to other regions (*F*(2,26) = 5.512, *p* = .026) (the region difference was slightly greater in controls but there was no region × diagnosis interaction). No significant differences between regions were found within subjects for FA, MD, PerpPD, or ParlPD.

**Table 2:** Mean values for histological variables for each region in MS brains. *SD* are given in brackets

Regions	Minicolumn width (μm)	Minicolumn spacing (μm)	Minicolumn Core width (μm)	Minicolumn microsegment number/mm^2^	Cell density	Axon bundle spacing (μm)	Axon bundle width (μm)
Area 9	37.7 (2.50)	15.5 (2.07)	28.9 (4.16)	203.5 (103.66)	123.1 (42.53)	45.3 (3.74)	8.2 (1.33)
Area 41	33.7 (4.14)	16.1 (1.10)	27.8 (2.61)	203.4 (40.56)	146.3 (49.82)	48.3 (6.82)	9.6 (0.70)
V1	27.1 (3.38)	15.5 (1.59)	26.7 (3.33)	247.7 (64.39)	95.8 (48.06)	28.6 (3.94)	7.3 (0.84)

Abbreviation: MS, multiple sclerosis.

**Table 3:** Mean values for diffusion measures for each region in MS brains and controls. *SD* are given in brackets

	Regions	FA	MD	AngleR	PerpPD	ParlPD
MS cohort	Area 9	0.0712 (0.02)	0.320^a^ (0.09)	0.898^a^ (0.09)	0.163^a^ (0.05)	0.334^a^ (0.24)
	Area 41	0.0909 (0.02)	0.435^a^ (0.07)	0.868^a^ (0.15)	0.179^a^ (0.20)	0.101^a^ (0.03)
	V1	0.0875 (0.03)	0.251^a^ (0.05)	0.911^a^ (0.13)	0.139^a^ (0.04)	0.113^a^ (0.04)
HC cohort	Area 9	0.0925 (0.02)	0.190 (0.03)	0.679 (0.02)	0.075 (0.02)	0.138 (0.02)
	Area 41	0.1018 (0.02)	0.120 (0.01)	0.677 (0.03)	0.057 (0.01)	0.081 (0.02)
	V1	(0.0916) (0.03)	0.164 (0.03)	0.821^b^ (0.06)	0.073 (0.01)	0.085 (0.03)

Abbreviations: FA, fractional anisotropy; HC, healthy control; MD, mean diffusivity; MS, multiple sclerosis.

a Value significantly higher than HC in between group comparison.

b Value significantly higher than other regions in within group comparison.

### 3.3 Histology differences between brain regions

Repeated measures ANOVA revealed a significant main effect of region on all histological measures (Tables 2 and 3 and Figure 4). Primary visual cortex had the narrowest minicolumns and narrowest axon bundles, with Area 41 having the widest spacing of axon bundles and the widest bundles (Table 4).

**Table 4:** Overall region differences for histology measurements in the MS brains determined by repeated measures ANOVAs are reported in the first row (effect of region). Post hoc *t*‐statistics are reported in the subsequent rows for specific region comparisons

Region	Minicolumn width (μm)	Minicolumn spacing (μm)	Minicolumn core width (μm)	Minicolumn microsegment number/mm^2^	Cell density	Axon bundle spacing (μm)	Axon bundle width (μm)
Effect of region	*F*(_2,14_) = 22.523 *p* < .001*	*F*(_2,14_) = 0.257 N.S.	*F*(_2,14_) = 0.440 N.S.	*F*(_2,14_) = 0.479 N.S.	*F*(_2,14_) = 2.493 N.S.	*F*(_2,16_) = 45.076 *p* < .001**	*F*(_2,16_) = 18.345 *p* < .001**
Area 9 vs. Area 41	*T* = 2.189 *p* = .065	–	–	–	–	*T* = −1.125 *p* = .293	*T* = −3.586 *p* = .007**
V1 vs. Area 41	*T* = −4.299 *p* = .004**	–	–	–	–	*T* = −7.340 *p* < .001**	*T* = −6.559 *p* < .001**
Area 9 vs. V1	*T* = 9.013 *p* < .001**	–	–	–	–	*T* = 18.149 *p* < .001**	*T* = 2.228 *p* = .056

Abbreviations: ANOVA, analysis of variance; MS, multiple sclerosis.

### 3.4 Relationship between histology measures

**Figure 5:**
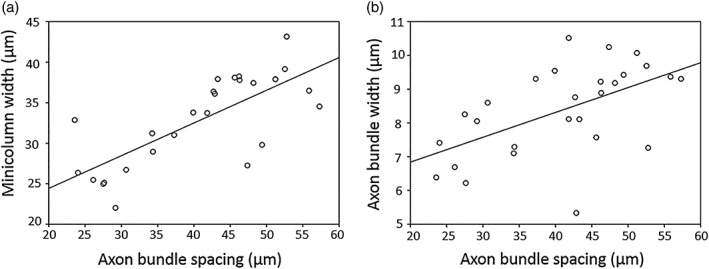
Relationships between histological measures of cortical cytoarchitecture in MS brains, pooled across regions

A strong positive correlation was observed between the width of the minicolumns in the cortex, as assessed by cell bodies, and the spacing of myelinated axon bundles (*r* = .718, *p* < .001, *p*_FDR_ = .000) (Figure 5). Bundle width also showed a positive correlation with bundle spacing (*r* = .548, *p* = .003, *p*_FDR_ = .0045) but the relationship between bundle width and minicolumn width assessed by cell bodies was not significant (*r* = .248, *p* = .222).

### 3.5 Relationships with clinical variables

**Figure 6:**
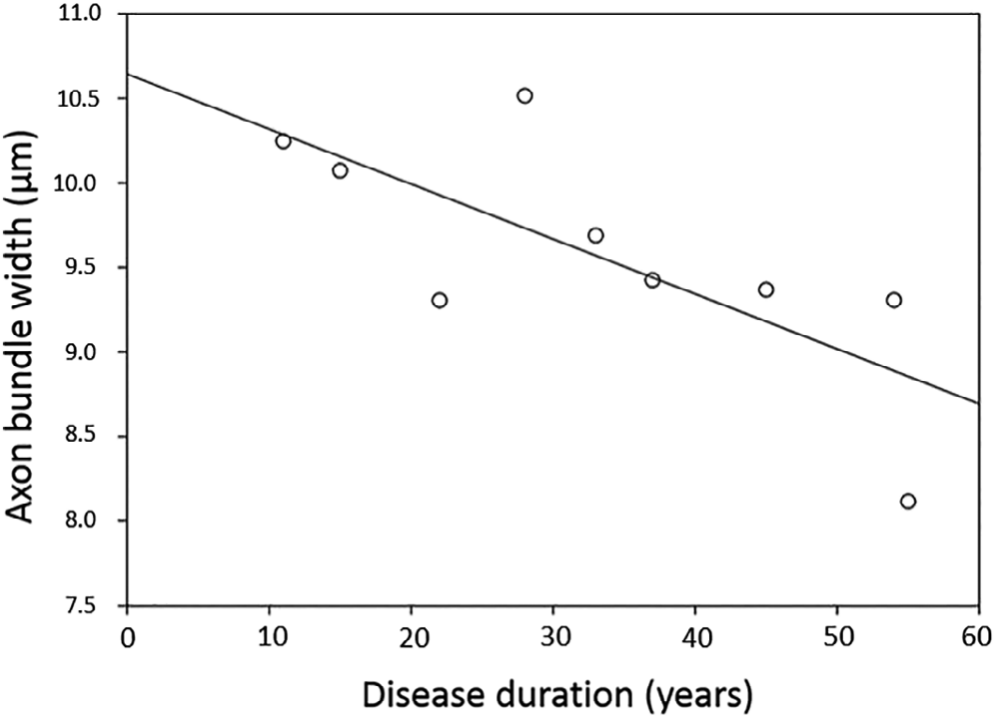
Relationship between bundle width and disease duration in primary auditory cortex (Area 41) in MS brains

Due to the presence of a strong correlation between disease duration and age (*r* = .883, *p* = .002), partial correlations controlling for age were used to investigate the relationships with disease duration. A significant negative correlation was observed between bundle width and disease duration in Area 41 (*r* = −.867, *p* = .011) (Figure 6) but not Area 9 (*r* = −.438, *p* = .278) or V1 (*r* = −.077, *p* = .856). Despite the correlations between axon bundle features and DTI measures reported in Table 5, relationships between the DTI measures and disease duration failed to reach significance.

**Table 5:** Relationships between diffusion metrics and cortical histological measures in MS brains. All *p* values were adjusted with FDR correction (FDR <0.05; *n* = 90 [five diffusion metrics × six histological measures × three brain areas]). Significant comparisons before or after FDR correction (*p* < .05 and *p*_FDR_ < .05) are shown in bold

Diffusion metric	Histological measure	BA9			BA41			V1		
		*r*	*p*	*p*_FDR_	*r*	*p*	*p*_FDR_	*r*	*p*	*p*_FDR_
AngleR	Minicolumn width	.912	**.001**	**.030**	.817	**.013**	.1671	−.578	.103	.4414
	Neuropil spacing	.773	**.024**	.24	.833	**.01**	.15	−.398	.288	.7482
	Core width	.879	**.002**	**.045**	.849	**.007**	.126	.387	.304	.76
	Microsegment number	−.654	.056	.336	−.786	**.021**	.2362	.548	.127	.4762
	Bundle spacing	.937	**.000**	**0**	.069	.859	.9677	−.603	.086	.4168
	Bundle width	.192	.621	.9267	.179	.645	.9267	−.691	.054	.336
PerpPD	Minicolumn width	.949	.949	.9677	−.664	.073	.3864	.131	.737	.9475
	Neuropil spacing	.778	.778	.9677	−.469	.241	.7054	−.138	.723	.9430
	Core width	.591	.591	.9267	−.557	.152	.5472	.038	.923	.9677
	Microsegment number	.515	.515	.9069	.696	.051	.336	−.102	.794	.9677
	Bundle spacing	.659	.659	.9267	−.068	.863	.9677	.297	.438	.8628
	Bundle width	.878	.878	.9677	.347	.361	.8517	.548	.126	.4762
ParlPD	Minicolumn width	.142	.716	.9430	−.357	.386	.8517	.415	.266	.7481
	Neuropil spacing	−.190	.625	.9267	−.296	.477	.8797	.139	.721	.9430
	Core width	.184	.635	.9267	−.252	.547	.9116	.233	.545	.9116
	Microsegment number	−.071	.856	.9677	.355	.388	.8517	−.377	.317	.7710
	Bundle spacing	.139	.721	.9430	.307	.422	.8628	.664	.051	.336
	Bundle width	−.089	.820	.9677	−.341	.369	.8515	.911	**.001**	**.030**
FA	Minicolumn width	.180	.643	.9267	.639	.088	.4168	−.397	.291	.7482
	Neuropil spacing	.295	.441	.8628	.707	.050	.336	−.272	.479	.8798
	Core width	.034	.930	.9677	.675	.067	.3768	−.398	.289	.7482
	Microsegment number	−.434	.243	.7054	−.588	.125	.4762	.436	.240	.7054
	Bundle spacing	−.048	.903	.9677	.246	.524	.9069	−.484	.187	.63
	Bundle width	.090	.817	.9677	.482	.189	.63	−.287	.455	.8712
MD	Minicolumn width	−.162	.678	.9387	−.032	.941	.9677	.310	.417	.8628
	Neuropil spacing	−.223	.563	.9212	−.284	.496	.8928	.057	.884	.9677
	Core width	.029	.942	.9677	.051	.905	.9677	.173	.657	.9267
	Microsegment number	.443	.233	.7489	.009	.984	.984	−.319	.402	.8614
	Bundle spacing	.021	.957	.9677	.204	.598	.9267	.592	.093	.4185
	Bundle width	.045	.909	.9677	−.049	.901	.9677	.696	.051	.336

Abbreviations: FA, fractional anisotropy; FDR, false discovery rate; MD, mean diffusivity.

## 4 DISCUSSION

The present study indicates the presence of correlation between the anisotropic diffusion observed in the cortex and microstructural properties of the cortex, such as minicolumnar and axon bundle organisation, which can only be studied with invasive histology. This suggests a potential role for diffusion MRI as a surrogate for microstructural changes in the cortex. It was explicitly not our goal to interpret the changes in diffusion MRI signal or provide a model for the deeper relationship with the underlying anatomy. Our aim in this work was to demonstrate that histological properties with known disease relevance correlate with diffusion‐based metrics, which may be useful as potential biomarkers. These act as surrogate measures for disease state and may possibly be a reflection of microanatomical alterations that are known to be of interest in neurodegenerative conditions.

Given previous histological findings of age‐ and pathology‐related changes in the cortex (Chance et al., 2006; Chance, Casanova, Switala, & Crow, 2008; Di Rosa et al., 2009; van Veluw et al., 2012), cortical diffusion surrogates could prove to be a powerful tool for investigating such changes without the destructive histological processing that limits tissue availability. It is therefore worth considering the feasibility of translating such markers into the in vivo clinical setting. The long acquisition times used in this study largely reflect the requirements of imaging PM tissue and are not necessary in vivo. Changes in tissue diffusivity and T2 cause an extremely low SNR regime so that achieving a diffusion‐weighted contrast comparable to in vivo scans requires long scan times (Miller et al., 2011) and alternative acquisition methods (Miller, McNab, Jbabdi, & Douaud, 2012). In vivo, the longer T2 and faster diffusion are much more conducive to imaging, and the primary challenge is to obtain sufficient SNR in a reasonable scan time. The diffusion scan SNR in the present PM study had an average SNR of 66.9 in B0 and 9.2 in *b* = 4,500 s mm^2^ volumes. Similar SNR values (and at least greater than an SNR of 2) are achievable with in vivo acquisitions. Sotiropoulos et al. (2013) reported SNR values of 9 for *b* = 1,000 at high resolution using the previous generation of magnets, therefore the latest systems will deliver substantial improvements; note that the reduced ADCs (and anisotropy) in the present study place it roughly in the same domain as *b* = 1,000 data in vivo (e.g., Miller et al., 2011). The necessary spatial resolution for in vivo cortical analysis (~1 mm) is also achievable; for example, strong gradients and simultaneous multislice imaging in the Human Connectome Project has enabled 1.25 mm, whole‐brain DTI acquisitions (Uğurbil et al., 2013) that clearly demonstrate cortical anisotropy (Sotiropoulos et al., 2013). Several studies have achieved sub‐millimetre resolution using ultrahigh field strengths (Dumoulin, Fracasso, van der Zwaag, Siero, & Petridou, 2018; Heidemann et al., 2012) and non‐EPI acquisition schemes (Sarlls & Pierpaoli, 2009; Setsompop et al., 2018). Sufficient resolution has been achieved to enable similar diffusion analysis in the cortex in vivo with realistic acquisition times (Anwander et al., 2010; McNab et al., 2013). It will be necessary to take account of potential movement artefacts and we would expect that sophisticated methods for detecting and correcting for motion will likely be a key factor in future studies (e.g., Andersson, Graham, Zsoldos, & Sotiropoulos, 2016). Adaptation of our analysis for in vivo use would allow longitudinal investigation with potential prognostic and diagnostic value.

### 4.1 Diffusion as an index of histology

Multiple anatomical correlations were found with the cortical diffusion signal—in particular, AngleR was found to correlate sensitively with changes in minicolumn width and axonal bundle characteristics. Due to the relatively small sample size, a common challenge in PM studies of this kind, it can be difficult to reliably detect relationships between variables. Some individual data points may have a strong influence on the estimated effect sizes—for example, in the Area 9 results, it could be argued that data points appear to cluster with two subjects further from the others (Figure 3). However, correlations in this region were statistically robust and survived FDR correction for multiple comparisons. The association with histological measures suggests the possibility that our DTI correlates may be sensitive to alterations of minicolumnar organisation produced by neurodegeneration. Neuron loss is considered to be one of the most clinically relevant surrogate markers of disease progression (Fisher et al., 2002) and one of the causes of minicolumn disruption (Wegner et al., 2006). The increased AngleR value in multiple sclerosis patients compared with healthy controls describes an increased angle between the radial direction across the cortex and the PDD, perhaps due to minicolumn alteration.

The significant correlation between axon bundle spacing and AngleR, and between axon bundle width and ParlPD, may be due to the hindrance to water diffusion imposed by the axonal membranes and myelin sheath. However, it was not possible to determine in the present study whether increased bundle width reflected changes in the width of individual axons, number of axons in the bundle, packing density of the axons or thickness of myelin sheaths, and this is an area that has not been well documented in the literature. Cytoarchitectural evidence from monkey cortex suggests that with increasing axon diameter there is an increase in both myelin sheath thickness and number of lamellae surrounding the axon (Peters, Sethares, & Killiany, 2001). In this scenario, either increased width of individual axons or increased numbers of axons within a bundle could lead to wider axon bundles with greater numbers of coherently arranged membranes. It is unclear how this relates to the net effect of axon bundles, minicolumns and other components at the scale of the MRI voxel.

Depending on the relative changes in axon size, packing density, myelin thickness, and myelin layering in wider bundles, the diffusion across axons could change, resulting in the higher diffusion‐derived values (AngleR and PerpPD) observed in the present study.

Further investigations are needed to clarify the most important of these factors and the interactions between different anatomical components. For example, axon bundle width, axon bundle spacing, and minicolumnar organisation of cell bodies may be independent of the other properties described above and also correlate with diffusion properties in the cortex. Despite the uncertainty in the specific contributions of various histological features to the observed directionality of diffusion in the cortex, the correlations reported here indicate that cortical diffusion may serve as a useful marker for changes in cortical microanatomy particularly for clinical neuropsychology applications. Future work could also help to clarify whether observed differences in axon bundle widths reflect differences in the number of axons, individual axon widths or packing density of the axons within the bundle.

Another study has attempted to relate cortical DTI in PM tissue to histological features in humans, finding that areas with high FA were also the areas where visual assessment of histology indicated radial organisation (Huang et al., 2012). Although studies have demonstrated a close correspondence between the direction of diffusion in the cortex and the orientation of neuronal processes in animal tissue, for example, (Jespersen et al., 2012), few studies have attempted to correlate specific values of diffusivity in the cortex with quantitative measures of cortical histology in the adult human.

The present finding of a correlation between cortical architecture and values relating to the diffusion suggests that markers of this sort should be incorporated into future research alongside the more traditional values of FA and MD to provide a more comprehensive picture of changes occurring in the cortex. The absence of a statistically significant relationship between histology and MD in the present study does not preclude an influence of cortical organisation on such values (e.g., if this relationship was weaker, the statistical power here might not have been sufficient to detect them).

Therefore, it may be premature to seek a hypothesis under which changes in PDD are not accompanied by specific changes in MD. Furthermore, the finding that the values relating to the direction of diffusion in the cortex did not seem to vary with PMI and SI suggests diffusion direction is amenable to study in PM tissue. The absence of a clear correlation between overall myelin density and the DTI measures is different from the picture generally reported by studies of white matter (e.g., Beaulieu, 2002; Song et al., 2002). A straightforward relationship may not be expected in grey matter given the far more complex structure of the cerebral cortex and the lower amount of myelin compared with white matter. The influence of cellular components, extracellular matrix, and dendritic structure is likely to be greater in cortical grey matter, constituting multiple interacting microstructural boundaries independent of myelin level.

### 4.2 Regional differences

The difference between brain regions in cortical DTI characteristics and cytoarchitecture has potential clinical relevance as selective regional changes may be informative for investigating neurological disorders. The present study found regional differences in minicolumn width between Area 9 and V1 that are similar to those that have been well characterised previously (Casanova et al., 2008; van Veluw et al., 2012). In this study, Area 41 contained relatively wide minicolumns compared to Area 9 and V1, which may be explained by the age‐related minicolumn narrowing normally observed in regions other than Area 41 (Chance et al., 2006; van Veluw et al., 2012). Axon bundle spacing showed a similar pattern, confirming the consistency between cell body and axon‐bundle‐based columnar measurements. The width of the axon bundles also differed between regions. Although this measure is uncommon, one study examining axon bundles in Area 41 found similar widths to those reported here (Seldon, 1981).

The current findings of regional differences in the values of cortical diffusion are consistent with a previous study which found that MD was different in frontal areas compared to occipital areas (Jeon et al., 2012). The present study found regional differences in AngleR, in particular, a significant difference between Area 9, Area 41 (most radial), and V1 (most tangential). The more tangential diffusivity in V1 may be due to the presence of the stria of Gennari which is a myelinated tract running parallel to the cortical surface, roughly in the middle of the primary visual cortex. Area 41 has been shown to have strongly radially organised cytoarchitecture (Sigalovsky, Fischl, & Melcher, 2006; von Economo & Koskinas, 1925) and very directional diffusion (as suggested by high FA and low MD in the present study). Differences in diffusion metrics across various cortical regions (Anwander et al., 2010; Jeon et al., 2012; Kang et al., 2012; McNab et al., 2013) could be influenced by variation in the relative thickness of cortical layers, introducing different mixtures of tangential and radial diffusion.

Some previous studies of cortical diffusion have looked at the dot product between the PDD and the vector normal to either the cortical surface or intermediate surfaces calculated within the cortex (Anwander et al., 2010; McNab et al., 2013). The present study used a much smaller voxel size than previous work, which may contribute to the greater range of values found here. For areas such as V1, where a strong effect of cortical depth on angle values has been shown (Kang et al., 2012; Kleinnijenhuis et al., 2013; Leuze et al., 2011), there may also be differences between methods depending on the depth of sample points. Previous findings in Area 41 have been mixed, with McNab et al. (2013) highlighting Heschl's gyrus as having a notably tangential PDD. In contrast, Kang et al. (2012) noted that the poleward side of Heschl's gyrus displays particularly radial values. Our average across the entire cortical depth provides a good picture of the average orientation for a given region. Our analysis found more radial diffusivity on average, consistent with detailed anatomical assessments of Area 41 which have found the majority of axons are oriented radially (Seldon, 1981), with well‐defined myelinated bundles (von Economo & Koskinas, 1925).

### 4.3 Relationships between histology measures

The present finding of a correlation between spacing of the minicolumns based on cell bodies and axon bundles is consistent with what is known about the structure of the minicolumn (Buxhoeveden & Casanova, 2002; Mountcastle, 1997) and previous work comparing the two measurements (Casanova et al., 2008).

In the present study, the width of bundles increased with spacing between the bundles, but it is not known whether this reflected a greater number of individual fibres within the bundle or less dense packing of the same number of fibres. Understanding this may shed light on the functional implications of such regional variation. It has been suggested that narrowly spaced minicolumns have more overlapping activations and function less independently (Chance et al., 2013; Harasty, Seldon, Chan, Halliday, & Harding, 2003). Those minicolumns may have fewer axons in their bundles due to the greater redundancy in their information output. This would be of particular relevance to demyelination and conditions of brain damage, but also disorders such as autism where one of the most prominent neuroanatomical hypotheses is concerned with altered minicolumn organisation (Casanova et al., 2002) and connectivity in fibre tracts (i.e., axon bundles) (Tommerdahl, Tannan, Holden, & Baranek, 2008).

### 4.4 Clinically relevant variation in measurements

Overall, the cortical DTI markers showed a difference between controls and MS brains. Markers relating to myelinated components of the cortex may complement other methods for characterising brain regions, age‐related changes, and the detection of pathology, particularly in multiple sclerosis. Cortical demyelination has been suggested to account for the moderate correlation between white matter damage and cognitive impairment (Kutzelnigg & Lassmann, 2006). Furthermore, changes in diffusivity in grey matter have been found to relate more closely to clinical measures than either changes or lesions in normal appearing white matter (Vrenken et al., 2006).

It is likely that the values derived from the diffusion signal are sensitive to a combination of features within the cortical tissue, including cell membranes associated with synapses, neurites, and cell bodies, and myelin associated with axons. By contrast, the different histological stains and individual histological measurements are constrained to assess only one feature at a time. This may explain the stronger diagnostic group difference detected by the DTI correlates compared with the individual histology measures.

The only relationship between disease duration and bundle width in histology was found in Area 41. Although this is not sufficient evidence to conclude that multiple sclerosis demyelination across the duration of disease influences bundle width, it is interesting that Area 41 may be more vulnerable due to its location deep in the Sylvian fissure next to the insula, as the demyelinating effects of cortical lesions have been shown to be more common in sulci and deep in‐foldings of the cortical surface, particularly in the insula (Kutzelnigg & Lassmann, 2006).

### 4.5 Limitations

The current study has been limited to investigating three regions of the cortex (Area 9, V1, and Area 41) due to the time‐consuming nature of the manual steps involved in both histological and MRI analysis. Future studies across the whole brain would depend on development of robust, automated image analysis tools for segmentation and registration of PM data, as well as advances in histological processing hardware.

PM tissue is known to show altered diffusion properties (Miller et al., 2011; Shepherd, Thelwall, Stanisz, & Blackband, 2009). Studies examining these changes in WM have demonstrated reductions in both FA and MD, for example, (Miller et al., 2011; Schmierer et al., 2008), but this has been much less studied in GM. Cortical slices of fixed rat brain have shown increases in extracellular apparent diffusion coefficient and apparent restriction size, with evidence of increased membrane permeability (Shepherd et al., 2009). Future work would also benefit from broadening the acquisition to acquire either a range of diffusion times or *q*‐values (i.e., multishell acquisitions).

McNab et al. (2013) demonstrated a high degree of radiality in primary motor cortex for both in vivo and PM tissue. It has been suggested that diffusion may be more tangential in somatosensory cortex but this was not so uniformly replicated in the portmortem tissue. Some studies, (e.g., D'arceuil & de Crespigny, 2007; Miller et al., 2011), have reported an influence of PMI (time between death and fixation) on diffusivity measures although other studies have suggested that these remain relatively constant after fixation, for example, (Kim, Zollinger, Shi, Rose, & Jeong, 2009). It is worth noting that tissue volumetric change due to fixation (i.e., shrinkage) stabilises within a few weeks (Quester & Schröder, 1997) and all of the cases in this study had been fixed for little more than the 3 years that Dyrby et al (2011) suggest is the initial period of stable SI. In testing for correlations in the present study, only FA showed a relationship with SI.

The effect of PMI on diffusivity values in the cortical grey matter are likely to differ from those in WM due to cellular components as well as the fixation process itself. Immersion fixation causes the GM to come into contact with the fixative immediately whereas it has to penetrate through to the WM, effectively resulting in a more extended PMI. Overall, there are reasonable grounds to expect a correspondence between cortical diffusivity assessments obtained in vivo and those obtained from PM tissue, although future research should focus on clarifying this. The full range of factors influencing diffusion in the cortex is not fully understood and other factors such as the packing density of axons and other membranes, and the relative volume fractions of these components may also contribute.

## 5 CONCLUSIONS

We describe an approach to the analysis of high‐resolution MRI diffusion data in the cortex that is sensitive to cytoarchitecture and myeloarchitecture in the human brain using DTI. Histologically measured widths of cell minicolumns and axonal bundles were correlated with direction of diffusion in the cortex. Further, we demonstrate regional differences in these aspects of cortical diffusion. There are many potential causes for these differences, and this study does not interpret the cause nor does it attempt to model the anatomy and its effects. We do observe consistent differences and correlations, and this is what we report here as we believe that these may be useful for developing biomarkers. The current work used MRI of PM tissue to enable the histological comparisons, but future application of this approach to sufficiently high‐resolution in vivo MRI scans could open up the possibility of detecting changes occurring in both normal and pathological development. The correlations with axonal bundles suggest that this technique may be of particular interest in demyelinating or connectional disorders, such as multiple sclerosis and autism.

## CONFLICT OF INTERESTS

R.M., K.M., M.T., M.J., and S.A.C. have submitted patent applications related to MRI analysis. S.A.C. is a cofounder of a company, Oxford Brain Diagnostics, from which he has received no funding towards the research or preparation of this manuscript. M.J. is a cofounder of a company, Oxford Brain Diagnostics, from which he has received no funding towards the research or preparation of this manuscript. No other conflicts of interest to declare.

## Data Availability

The data that support the findings of this study are available from the corresponding author upon reasonable request.
